# Do Red Deer Stags (*Cervus elaphus*) Use Roar Fundamental Frequency (F0) to Assess Rivals?

**DOI:** 10.1371/journal.pone.0083946

**Published:** 2013-12-30

**Authors:** Maxime Garcia, Benjamin D. Charlton, Megan T. Wyman, W. Tecumseh Fitch, David Reby

**Affiliations:** 1 Department of Cognitive Biology, University of Vienna, Vienna, Austria; 2 School of Psychology, University of Sussex, Brighton, United Kingdom; German Primate Centre, Germany

## Abstract

It is well established that in humans, male voices are disproportionately lower pitched than female voices, and recent studies suggest that this dimorphism in fundamental frequency (*F0*) results from both intrasexual (male competition) and intersexual (female mate choice) selection for lower pitched voices in men. However, comparative investigations indicate that sexual dimorphism in F0 is not universal in terrestrial mammals. In the highly polygynous and sexually dimorphic Scottish red deer *Cervus elaphus scoticus*, more successful males give sexually-selected calls (roars) with higher minimum F0s, suggesting that high, rather than low F0s advertise quality in this subspecies. While playback experiments demonstrated that oestrous females prefer higher pitched roars, the potential role of roar F0 in male competition remains untested. Here we examined the response of rutting red deer stags to playbacks of re-synthesized male roars with different median F0s. Our results show that stags’ responses (latencies and durations of attention, vocal and approach responses) were not affected by the F0 of the roar. This suggests that intrasexual selection is unlikely to strongly influence the evolution of roar F0 in Scottish red deer stags, and illustrates how the F0 of terrestrial mammal vocal sexual signals may be subject to different selection pressures across species. Further investigations on species characterized by different F0 profiles are needed to provide a comparative background for evolutionary interpretations of sex differences in mammalian vocalizations.

## Introduction

A key objective of research in animal vocal communication is to identify the origin, nature and function of the information contained in acoustic signals, in order to understand how selection pressures have shaped their evolution [1]. Sexually-selected male calls are displays typically given during the reproductive period [2]. In vertebrates, these calls are often multi-component signals [3], [4], which encode information on long term, static (size [5]–[11]; sex [9], [12]; identity [13]–[17]) or shorter term, dynamic (arousal, 'motivational' state [18]–[20]; hormonal levels [8], [21], [22]; physical condition [20], [23]) attributes of callers. Studies of these calls in terrestrial mammals indicate that this information may be used in both male competition and/or female mate choice contexts in a wide range of species (baboon: [24], bison: [11], fallow deer: [7], orangutans: [25]) with a handful of experimental studies actually demonstrating such a function (koala: [26], red deer: [27]–[29]).

The generalization of the source-filter theory of voice production [30] to non-human vocal signals [31]–[33] has led to considerable advances in our understanding of the acoustic structure of mammalian calls (reviewed by Taylor & Reby, [34]). According to this theory, voiced vocalizations result from a two-step production process. First, a source signal (the glottal wave) is generated by vibrations of the vocal folds in the larynx. When these vibrations are periodic, their rate determines the fundamental frequency (F0), and the perceived pitch of the radiated vocalization. This glottal wave subsequently travels through the supra-laryngeal cavities of the vocal tract that act as a filter, adding broadband resonance frequencies or ‘formants’ to the spectral envelope of the emitted vocalization. Because F0 and formants are produced independently, and both subject to various biomechanical constraints, both parameters have the potential to carry reliable information about the signaler [34], [35].

The function of formants in sexually-selected male calls has recently received considerable attention, with studies identifying negative correlations between formant frequency spacing (a measure of formant scaling in the frequency domain) and body size in marsupials (koala: [10]), ungulates (red deer: [5], fallow deer: [7], bison: [11]), carnivores (elephant seals: [6], giant panda: [9]), and primates (macaques: [33], colobus monkeys: [36]), due to the allometric relationship between formants, vocal tract length (VTL), and overall body size [33], [35]. In contrast, research on the determination and function of F0 variation is less conclusive. A negative correlation between body size and mean F0 is expected across a wide range of mammal species (e.g. [37], [38]) because animals with larger and heavier vocal folds should produce calls with lower F0 [39]. However F0 is typically poorly related, or not related at all, to body size variation within multiple species of adult mammals (baboons: [40], fallow deer: [7], humans: [41], Japanese macaques: [42], lions: [43], red deer: [5]; but see [9], [44], [45]).

Deer have proved to be a very useful model for testing hypotheses on the variation and function of spectral components in sexually-selected male vocalizations. During the breeding season (or rut), red deer (*Cervus elaphus*) stags give loud, conspicuous roars [46]. While roaring, stags pull their larynx down towards the sternum, thereby extending their vocal tract and lowering the formants (or vocal tract resonances) of their roars [47], [48]. The minimum formant frequency values and spacing, achieved when the larynx is fully retracted to the sternum and the vocal tract fully extended, provide reliable cues to body size [5], [47] which are attended to by both male and female conspecifics: resynthesized roars with formants indicative of larger individuals elicit stronger responses from potential male rivals [27] and are preferred by oestrous females [49].

Although F0 is a highly salient and individually distinctive feature of roars [50], it is unlikely to function as an index of body size, because neither vocal fold length nor F0 are correlated with body weight in adult stags [5], [48]. However recent playback experiments indicate that red deer hinds prefer male roars with relatively high F0 [29]. While the communicative value of F0 in red deer roars remains unclear, it has been suggested that it may function as an index of subglottal pressure [51], with high F0s associated with increased activity, arousal or superior physical condition [5], [52], [53].

In order to investigate the hypothesis that roar F0 plays such a role in the context of male-male competition in red deer, we conducted playback experiments testing the reaction of harem-holding adult male red deer to re-synthesized stimuli with different median F0s, in their natural environment, mimicking the intrusion of an unfamiliar adult male in the close vicinity. More specifically, we examined the behavioural response of stags to playbacks of roaring stimuli with median F0s of 70 Hz, 100 Hz, 130 Hz or 160 Hz. We predicted that stags would respond more strongly to roars characterized by higher F0 that may indicate more highly motivated, threatening opponents. While the effect of F0 on perceived attractiveness in male human voice has been thoroughly investigated using experimental approaches [54]–[56], to our knowledge the current study is the first playback experiment to investigate the potential function of F0 in the context of male competition in a non-human mammal.

## Materials and Methods

### Authorizations and Approvals

Permissions to run experiments and park off roads within Richmond Park were granted by Simon Richards, Park Superintendent of the Royal Parks of London. The playback protocol used in this experiment was reviewed an approved by the Ethical Review Committee of the University of Sussex (UoS ERC Reby/Wyman 20/10/09).

### Study Site and Animals

Playback experiments were conducted at Richmond Park, London, UK, during the 2012 autumn breeding season (from October 1^st^ until October 19^th^), where 8 resident red deer stags served as subjects. The red deer population at Richmond Park is semi-captive and culled on a yearly basis.

### Playback Stimuli

Red deer stags produce two distinct types of roars during the mating season: common roars and harsh roars [5]. The most frequent type, the common roar, is defined by a mostly tonal structure and slow amplitude onsets and offsets, as well as a pronounced formant modulation as the vocal tract is lengthened during the course of the vocalization [47]. Harsh roars are less frequent, and typically given after intense herding of females and/or during vocal contests with other males [5], [48]. They are defined by deterministic chaos (non-periodic vibrations of the vocal folds), abrupt amplitude onsets and offsets and reduced formant modulation as the vocal tract is fully extended before and throughout the vocalization [5]. Because harsh roars typically do not have a discernible fundamental frequency [5], common roars were used as stimuli in our playback experiments.

**Figure 1:**
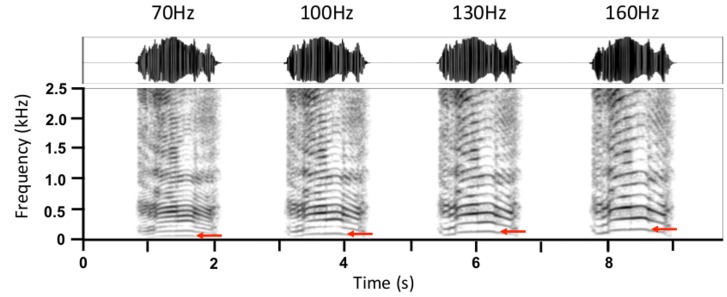
Spectrograms of resynthesized roars from one of the male exemplars showing the four F0 variants. F0 (indicated by the red arrow) was re-scaled to median values of 70 Hz, 100 Hz, 130 Hz and 160 Hz. All other acoustic parameters (duration, intensity contour, amplitude, formant frequencies etc.) remained unchanged.

The common roars that were used to create the playback stimuli were recorded by DR from 7 farmed adult Scottish red deer stags (*Cervus elaphus scoticus*) at Redon experimental farm in France and in New Zealand farms. These stags were comparable in body size to adult stags found in Richmond Park, and were unfamiliar to the tested stags. Our stimuli were resynthesized using the Pitch Synchronous Overlap and Add (PSOLA) [57] algorithm in Praat (version 5.3.12, [58]). PSOLA enables the independent modification of the fundamental frequency (F0) whilst leaving all other acoustic features unchanged (see Fig. 1). The median F0 of the roar stimuli was set at 70 Hz, 100 Hz, 130 Hz and 160 Hz using the “change gender” command in Praat and the following settings: pitch floor  = 30 Hz, pitch ceiling  = 300 Hz, formant shift  = 1, pitch range factor  = 1 and duration factor  = 1. These values cover the range of F0 observed in farmed and free-ranging populations of Scottish red deer stags (see [5]; range  = 66 Hz to 168 Hz, average  = 112 Hz) and have already been shown to elicit preferences in oestrous hinds in previous experiments [29]. After the re-synthesis procedure, the maximum amplitude of each bout was normalized to 99% peak (using Audacity version 2.0.1).

A total of 28 different playback sequences were created (4 fundamental frequency variants for each of the 7 exemplar stags). Each playback sequence consisted of two roar bouts (based on exemplars produced by the same stag), separated by 20 seconds of silence. Bouts were composed of 1 to 3 different roars (average ± SD  = 2.06±0.56) and thus lasted between 2.06 and 8.72 seconds (average ± SD  = 4.39±1.51 s), representing the natural variation of this parameter [5].

### Playback Design and Procedure

Each of the 8 focal stags were presented with all 4 F0 variants from the same exemplar, resulting in a total of 32 playback experiments (i.e. a total of four playbacks per stag). Using re-synthesis enables us to present individual stags with several F0 variants from each of the exemplars, ensuring that only F0 varies between the four stimuli presented to one given stag. It also enables us to present different exemplars to different focal stags, thereby maximizing the external validity of our observations by ensuring that our stimuli cover the natural variability of roars. In other words, re-synthesis preserves independence between F0, the parameter of interest, and other untouched acoustic parameters (e.g., duration, formant frequency, F0 contour, intensity contour etc.). The variation in these unmodified parameters is fixed (and presumably inter-dependent) within subjects, and represents the natural variation between subjects. Presentation order was alternated using a Latin square design. One of the exemplars was used twice in the experiment (played to two different stags). To prevent stags from habituating to the playback procedure, sequences were played back a minimal of 2 hours apart, and a maximum of 3 sequences were presented to each stag on a given day (a level of encounters consistent with the size and density of the red deer population at Richmond Park, where stags are likely to interact with several intruders within a day during the rut – pers. obs.).

Playback trials were conducted throughout the day when a stable harem was located (defined as a mature stag and at least 4 hinds (number of hinds average ± SD  = 17.63±9.62)). The focal stag had to display an overall behaviour typical of the rutting period (e.g., some roaring activity, herding and defense against other males). Playback trials were initiated when the harem holder was not engaged in a direct interaction with another stag, and not disrupted by new hinds entering the harem at least for the 15 minutes preceding the playback sequence. The experimenter placed a loudspeaker (Anchor Audio Liberty 6000HIC) 40–70 m away from the stag at an amplitude of 98 dB, measured at 1 m away from the source. The loudspeaker was connected to a MacBook Pro (Mac OS X, version 10.7.4) using a 50-m co-axial cable. This allowed the experimenter to initiate playback sequences while standing perpendicular to the “Stag-Loudspeaker” axis, and in doing so, minimize disturbance around the speaker position. The focal stag’s response was recorded using tripod-mounted Sony (HDR TG3) or Canon (LEGRIA FS200) video camcorders, from the beginning of the sequence until 5 minutes after the last bout terminated.

### Behavioural and Statistical Analyses

The video sequences were analyzed frame-by-frame (frame  = 0.04 s) using Gamebreaker v7.5.5 software (SportsTec, Sydney), starting when the first bout was initiated, until 5 minutes after the second bout was terminated. To quantify the behavioural responses of subjects to our stimuli we measured the number of common roars (CR), number of harsh roars (HR), time spent looking towards the speaker (LK), time spent walking towards the speaker (WK), latency to look towards the speaker (LTLK), and latency to roar back (LTR).

Behaviours were characterized as “oriented towards the loudspeaker” when the direction (looking/walking) was judged to be at a maximum of 30 degrees from the stag-loudspeaker axis. The behavioural responses were measured by MG. An independent observer double-coded 10% of the trials. The inter-observer agreement of 100% confirmed the reliability of the coding procedure.

Because the response variables were not normally distributed (Kolmogorov-Smirnov test: P<0.05), with the exception of CR (P  = 0.181), we used non-parametric Friedman tests (with exact statistics, appropriate to our sample size [59]) to examine the effect of the F0 variant and order of presentation on each of the dependent variables characterizing the stag’s behavioural response. P-values were corrected for multiple testing following Benjamini & Hochberg [60]. All the statistical tests were computed using SPSS v.19, significance levels were set at p  = 0.05 and two-tailed statistics are reported.

## Results

**Figure 2:**
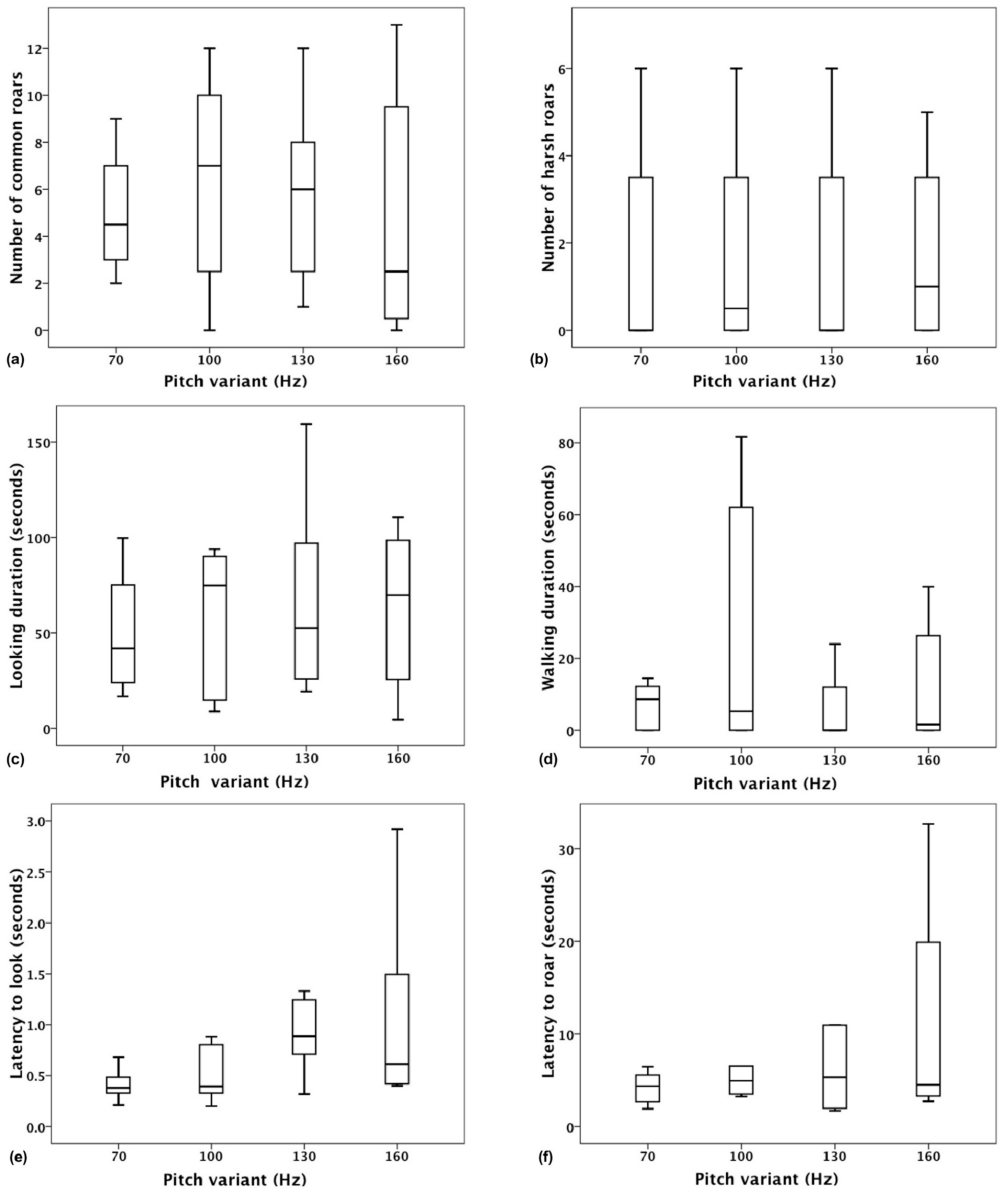
Behavioural responses of focal stags to playback experiments. Boxplots (with first, second (median), third quartiles, and range; outliers are not represented) illustrating the effect of F0 variant on the key behavioural variables characterizing the stag's response to playbacks (Friedman test, p-values adjusted following Benjamini-Hochberg correction; N  = 8); number of common (A) and harsh (B) roars, time spent looking (C) and walking (D) towards the loudspeaker, latency to look at the speaker (E) and latency to roar back after stimulus presentation (F).

During the playback experiments, males typically interrupted their current behaviour by looking (100%; n  = 32/32 trials) and roaring back (94%; n  = 30/32 trials) at the loudspeaker. Stags also moved towards the loudspeaker in half of the trials (50%; n  = 16/32). Raw scores for all the response variables across the 32 playback trials are reported in Table S1. Friedman comparisons with Benjamini-Hochberg correction for multiple testing showed that the effect of presentation order was non significant for all the tested variables (all P>0.05), indicating that stags did not significantly habituate across the presentation of the four F0 variants. Friedman comparisons with Benjamini-Hochberg correction for multiple testing showed that the F0 variant did not have a significant effect on any of the response variables (with corrected p-values: CR: Chi-Square (N  = 8, df  = 3)  = 2.520, P  = 1; HR: Chi-Square (N  = 8, df  = 3)  = 1.056, P  = 0.974; LK: Chi-Square (N  = 8, df  = 3)  = 0.750, P  = 0.890; WK: Chi-Square (N  = 8, df  = 3)  = 1.544, P  = 1; LTLK: Chi-Square (N  = 8, df  = 3)  = 9.150, P  = 0.132; LTR: Chi-Square (N  = 6, df  = 3)  = 1.400, P  = 1; see Fig. 2). Finally, a separate test confirmed that F0 variant did not have an effect on the cumulated number of Common Roars and Harsh Roars (CR+HR: (Chi-Square (N  = 8, df  = 3)  = 0.5, P  = 0.930)).

## Discussion

We found that the strength of the agonistic response of free-ranging adult Scottish red deer stags to playbacks of re-synthesized roars was not affected by the F0 of the roar. Neither the vocal response (latency and number of roars) nor the approach response (walking towards speaker) nor the stag’s attention (latency to look and looking duration) differed between F0 variants. This is in contrast with similar experiments testing the effect of formant frequencies in red deer stags, where roars with lower formant frequencies (indicating larger individuals) provoked stronger responses from focal stags [27]. It is important to acknowledge that our results are based on observations involving a relatively small number of focal stags, raising the possibility that our design may lack statistical power to highlight very small-sized effects. However we did not identify any noticeable trend on any of the response variables across the F0 variants (with the exception of latency to look, which was significant (P  = 0.022) prior to correction for multiple testing). While further investigations involving a larger sample size may be required to investigate the possibility of very small effects of pitch variation on stag’s reaction, the present results enable us to exclude a strong role for roar F0 in determining the dynamic of male agonistic interactions. This observation is consistent with anatomical and acoustical data in this species: in adult male Scottish red deer, neither vocal fold length [48] nor F0 [5] are correlated with adult body size, a key factor in determining the outcome of male contests [61]. Previous studies that have investigated the role of roaring during intrasexual competition have shown that stags assess the fighting ability of their opponents using roaring rate [62] as well as body size using the roar's vocal tract resonances (formants) [27]. Our results show that F0 in the roars of intruding red deer stags does not play an equivalent role.

Studies of acoustic variation and playback experiments have confirmed the role of F0 as an index of body size in male competition in several groups of vertebrates (frogs [63], [64], birds [65] and mammals [9], [44]). However, male bullfrogs (*Rana catesbeiana*) do not respond differently to F0 variation in rival male vocalizations despite strong negative correlations between F0, body size and associated fighting ability [66]. In contrast, in many mammal species, including humans, F0 is not significantly correlated with adult body size (baboons: [40], fallow deer: [7], humans: [41], Japanese macaques: [42], crested macaques: [67], lions: [43], red deer: [5], koalas: [10]). However, F0 appears to convey useful non-size information across a wide range of vertebrate species: established dominance in fallow deer [7] and crested macaques [67], perceived dominance in humans [68]–[70], hormonal state in humans [71] and zebra finches [72], and resource-holding potential in several species of birds [73], [74].

In addition, human salivary testosterone is a negative predictor of F0 in males [71], [75], and males with lower pitch voices are perceived as more physically and socially dominant [69], more attractive [54]–[56] and better leaders [76], corroborating observations showing that human males with lower F0 appear to have higher mating [56] and reproductive [77] success. The positive effect of testosterone on vocal fold length (sheep: [78], humans: [75]), which consequently lowers F0 [39], is likely to be a key underlying cause of the negative correlation between maleness-related traits and F0.

A direct consequence of sexual selection for a lower F0 in male vocalizations is that in many species, including humans, males have a disproportionately lower F0 than females (baboons: [79], fallow deer: [15], lions: [43], humans: [80]). In contrast, in Scottish red deer, there is no sexual dimorphism in call F0 between males and females [29]. In fact, after correcting for body-size differences between the sexes, male red deer may have shorter vocal folds and higher pitched vocalizations than expected for their size, relative to females (Reby, unpublished data). This suggests that, unlike antler strength, which is positively correlated with testosterone levels [81], vocal fold length and F0 may be independent of androgen levels in Scottish red deer. These observations are consistent with the documented positive correlation that exists between male roaring minimum F0 and reproductive success in this subspecies [5]. Moreover, while experiments investigating responses to F0 variants in perioestrous females failed to identify differential responses [82], [83], a study carried out on oestrous females highlighted their preference for higher pitched roars [29]. Besides, harems are very unstable [46], [84] and females often leave their current harem to visit other males when in oestrus [84]. Altogether, this suggests that in Scottish red deer, the relatively high F0 of male roars may be a consequence of intersexual, rather than intrasexual, selection.

More generally, polygynous deer are characterized by strong interspecific variation in the F0 of male sexually-selected vocalizations [48], which is clearly independent of interspecific variation in body size. Fallow deer (*Dama dama*), Corsican deer (*Cervus elaphus corsicanus*), and Japanese sika deer (*Cervus nippon nippon*), three polygynous species smaller in size relative to red deer, illustrate both interspecific and intraspecific F0 variation: fallow deer males have a large descended larynx and produce an almost infrasonic low pitched groan, with lower F0 (F0mean  = 28.2 Hz, [15]) than females (F0mean  = 365 Hz, [85]), both sexes of Corsican deer produce very low-pitched calls (F0mean  = 40.1 Hz for male calls and 86.7 Hz for female calls, [86]), and male sika deer produce a high-pitched whistle with higher mean F0 than females (mean F0 = 1187 Hz for males and 968 Hz for females, [87]). This variation, both in terms of range, and in terms of the direction of sex dimorphism, suggests that very different selection pressures operate on the fundamental frequency of sexually-selected calls in polygynous deer.

It has been proposed that high F0 calls may be indices of physical capacity [5], [52], [53]. Indeed the production of higher frequencies may require higher subglottal pressure [51], [88] and stronger muscular contraction [53], and might therefore provide relevant information on the caller’s condition. High frequency signals have also been correlated with improved glottal efficiency and radiation [53], which could in turn improve the active space of the vocalization and consequently increase mating opportunities for males that produce them. Nevertheless, a high fundamental frequency will reduce the density of the harmonics sampling the formant envelope, decreasing the salience of the formant structure [89], potentially affecting the ability of the signal to broadcast size-related information in formant frequencies [83]. Finally, independently of its causes, it is unclear how selection for high F0 in male vocal sexual signals is compatible with a function of F0 as an index of androgen levels in the same species.

In conclusion, our results have shown that the strength of Scottish red deer stags’ agonistic responses to roars of simulated intruders is not affected by the F0 of the re-synthesized roar. This suggests that F0 does not play a role in red deer male competition (at least in this subspecies) and that in the male-male context, size-related formant variation is the key information conveyed in the acoustic structure of roars. In order to better understand how sexual selection operates on the different spectral components of vocal sexual signals, leading to the extraordinary acoustic diversity observed in polygynous deer, future work should involve the playback of re-synthesized vocalizations investigating the propagation properties of source vs. filter components as well as their effect on male and female listeners in a wider range of species. Such insight would provide a very useful background for understanding the roles and evolutionary origins of these strongly sexually dimorphic components of the human voice.

## Acknowledgments

We thank the Royal Parks of London and Simon Richards for granting access to the deer and John Bartram, head Gamekeeper at Richmond Park, for facilitating our observations. We also thank Solène Derville for helping with double-coding the videos.

## Supporting Information

Table S1 **Independent and dependent variables for all 32 playback trials.**
(PDF)
