# Supplementary material for: Do Red Deer Stags (Cervus elaphus) Use Roar Fundamental Frequency (F0) to Assess Rivals?
Source: PLoS One. 2013 Dec 30;8(12):e83946. doi: 10.1371/journal.pone.0083946 (PMC3875517; doi:10.1371/journal.pone.0083946)
Supplement: Table S1 — Independent and dependent variables for all 32 playback trials. (PDF) [file pone.0083946.s001.pdf]

Focal Stag	Exemplar stag	F0 (Hz)	Order	Nb hinds/harem	CR	HR	LK (s)	WK (s)	LTLK (s)	LTR (s)
4	E3	70	3	20	5	0	20.17	0	0.32	3.73
4	E3	100	4	24	8	0	11.98	0	0.20	4.53
4	E3	130	1	28	9	0	75.99	0	1.33	1.70
4	E3	160	2	35	6	0	67.63	14.76	0.43	3.31
5	E1	70	1	11	9	0	50.58	12.72	0.34	1.95
5	E1	100	2	11	12	1	93.8	10.53	0.31	9.03
5	E1	130	3	10	12	0	25.99	0	0.73	6.46
5	E1	160	4	18	0	0	71.8	1.72	0.47	
9	Yellow991	70	3	25	3	0	47.36	11.68	0.49	43.19
9	Yellow991	100	4	30	0	0	8.84	0	0.39	
9	Yellow991	130	1	25	1	0	25.8	0	1.16	30.30
9	Yellow991	160	2	40	0	2	23.52	0	0.40	7.77
11	E2	70	4	11	2	1	251.44	14.48	0.21	5.02
11	E2	100	1	4	8	3	78.99	47.37	0.88	3.52
11	E2	130	2	5	4	1	19.36	0	1.04	3.53
11	E2	160	3	15	2	0	27.7	0	1.64	32.67
13	Blue328	70	2	19	4	6	27.84	6.72	0.68	1.92
13	Blue328	100	3	18	1	0	17.72	0	25.10	5.35
13	Blue328	130	4	19	1	0	29.2	0	0.32	1.98
13	Blue328	160	1	20	25	17	230.75	37.89	2.92	5.28
15	Red109	70	4	30	9	6	99.68	10.6	0.48	2.67
15	Red109	100	1	15	6	6	86.52	81.72	0.35	6.55
15	Red109	130	2	11	7	6	159.33	30.47	0.70	7.11
15	Red109	160	3	36	1	1	4.62	0	0.76	2.72
16	E4	70	2	10	3	0	16.88	0	0.36	5.56
16	E4	100	3	10	4	0	70.69	0	0.40	17.31
16	E4	130	4	8	7	6	91.67	24.01	0.72	10.95
16	E4	160	1	15	13	1	110.6	1.52	1.35	3.75
17	E1	70	4	20	5	0	36.72	0	0.40	6.48
17	E1	100	1	7	12	4	286	76.84	0.73	3.25
17	E1	130	2	7	5	0	102.32	0	2.83	31.91
17	E1	160	3	7	3	5	86.4	39.96	0.41	19.89
